# Evaluation of the Effect of Hydroethanolic Root Extract and Solvent Fractions of *Cyphostemma adenocaule* (Steud. ex A. Rich) Descoings ex Wild & Drummond on Cell-Mediated Immune Response and Blood Cell Count in Mice

**DOI:** 10.1155/2021/1838903

**Published:** 2021-02-10

**Authors:** Dehnnet Abebe, Gomathi Periasamy, Aman Karim, Helen Bitew

**Affiliations:** ^1^Debre Markos University, Debre Markos, Ethiopia; ^2^Mekelle University, Mekelle, Ethiopia

## Abstract

**Introduction:**

*Cyphostemma adenocaule* (Steud. ex A. Rich) Descoings ex wild & Drummond (Vitaceae) has been used in traditional medicine for the management of various immunological and hematological disorders in different areas of Ethiopia and the rest of the world. In Ethiopia, the plant is used for the management of enlarged spleen, rabies virus, helminthic infection, snake bite, and various types of tumors.

**Objective:**

To evaluate the effect of hydroethanolic root extract and solvent fractions of *Cyphostemma adenocaule* on cell-mediated immunity (delayed-type hypersensitivity), organ index (spleen and liver), and blood cell count in Swiss albino mice.

**Materials and Methods:**

Acute oral toxicity test was conducted using nulliparous and nonpregnant Swiss albino mice following OECD 425 limit test method. Delayed-type hypersensitivity model was used to evaluate the effect on cell-mediated immunity. The experimental animals were divided into twelve groups which were sensitized and challenged with sheep red blood cells on day 0 and day 7, respectively. Levamisole 50 mg/kg was used as stimulant control, whereas cyclophosphamide 30 mg/kg was used as suppressant control. Hydroethanolic root extract (100 mg/kg, 200 mg/kg, and 400 mg/kg), aqueous fraction (100 mg/kg, 200 mg/kg, and 400 mg/kg), and *n-*butanol fraction (100 mg/kg, 200 mg/kg, and 400 mg/kg) were administered for seven days. The paw volume was measured using a digital plethysmometer before challenge and 24 hours after challenge. Blood was collected, and organs (spleen and liver) were isolated from each challenged mouse to determine blood cell count and organ index, respectively.

**Results:**

No mortality and noticeable behavioral changes were observed among all mice receiving hydroethanolic root extract and solvent fractions at a dose of 2000 mg/kg. Hydroethanolic root extract and solvent fractions of *Cyphostemma adenocaule* showed enhancement of delayed-type hypersensitivity, organ index, and blood cell count. Hydroethanolic root extract at a dose of 400 mg/kg showed the highest and statistically significant stimulation of delayed-type hypersensitivity (0.123 ± 0.010) and blood cell count compared to the vehicle.

**Conclusion:**

Hydroethanolic root extract and solvent fractions of *Cyphostemma adenocaule* showed a stimulatory effect on cell-mediated immunity and hematopoiesis.

## 1. Introduction

Alteration of the structure and functions of the immune system leads to suppression or stimulation of the immune response. The immune system which can be innate or adaptive is responsible for the protection of the body from infection and other immunological disorders [1].

The whole plant and phytoconstituents of many plants of various taxonomic classes exhibited immunomodulatory properties to an antigenic material that gets access to the body of living cells. *Cyphostemma adenocaule* (Vitaceae family) is an herbaceous climber widely distributed in Ethiopia [2]. In traditional medicine, *C. adenocaule* has been used for the treatment of various illnesses such as malaria, helminthic infection, cancer, tumors, snake bite, rabies, and anthrax [3–6]. Earlier scientific studies showed that *C. adenocaule* has *in vitro* antiplasmodial [7], antioxidant [8], and deworming [9] effects. The anti-infective, antitoxin, and antitumor activity of this plant might be due to its effect on the cellular immune system and accessory cell types. Therefore, this study aimed to demonstrate the effect of hydroethanolic root extract and solvent fractions of *C. adenocaule* on cell-mediated immunity, blood cell count, and organ index in Swiss albino mice.

## 2. Materials and Methods

### 2.1. Materials

#### 2.1.1. Plant, Chemicals, Reagents, and Standard Drugs

The roots of *C. adenocaule *were collected from the Angacha riverside of Teda woreda, North Gondar, Ethiopia. The chemicals and reagents used for the study were purchased from local suppliers in Mekelle, Ethiopia. Normal saline (Addis Pharmaceutical Factory/APF, Addis Ababa, Ethiopia), *n*-hexane (Loba Chemie. Pvt. Ltd, India), ethanol (Fine Chemical general trading, Addis Ababa, Ethiopia), distilled water (Amshaj Manufacturing plc., Addis Ababa, Ethiopia), *n*-butanol (CARLO ERBA Reagents SAS, France), gum acacia (Shreeji Pharma International, India), levamisole (Ningbo Shuangwei Pharm. Co. Ltd., China), and cyclophosphamide (Cadila health care Ltd., India) were used. All the chemicals, reagents, and standard drugs were of laboratory/analytical grade. Sheep blood and Alsever's solution were also used during the study.

#### 2.1.2. Experimental Animal

Female nulliparous and nonpregnant Swiss albino mice aged 8–12 weeks were used for the acute toxicity study, whereas for the main study, healthy, Swiss albino mice of either sex (weight 25±5 g, and 6–8 weeks of age) obtained from the animal house of the School of Pharmacy, College of Health Sciences, Mekelle University, were used. The mice were housed in a laboratory of 12/12 hr light/dark cycle with the provision of a commercial pellet diet and water *ad libitum*. Before beginning the experiment, the animals were acclimatized to the laboratory condition for a week.

### 2.2. Methods

#### 2.2.1. Collection and Authentication of the Plant

The plant sample was collected from Angacha riverside and authenticated by Mr. Getnet Chekol (Botanist, University of Gondar, Ethiopia), and a sample specimen (voucher DA01/2018) was deposited in the herbarium of the university. The roots of the plant were collected, washed with tap water, and shade-dried.

#### 2.2.2. Extraction and Fractionation

Shade-dried roots were powdered with an electric grinder of which one kilogram of the powder was macerated with 70% (v/v) ethanol (1 : 5 ratios) for 72 hours which was then filtered with a muslin cloth and Whatman filter paper No. 1 to remove any insoluble matter.

The marc was remacerated twice with the same solvent for 72 hours [8]. All the filtrates were dried in an oven at 37°C. 60 gram of the dried extract was dispersed in water (1 : 5 ratios) and fractionated by successively treating the dispersed medium with *n*-hexane and *n*-butanol in triplicates. The three fractions (aqueous, *n*-butanol, and *n*-hexane) were dried in an oven at 37°C.

#### 2.2.3. Sheep Red Blood Cell (SRBC) Preparation and Standardization

Fresh blood was collected from the external jugular vein of sheep and mixed with freshly prepared Alsever's solution (2% dextrose, 0.8% sodium citrate, 0.055% citric acid, and 0.42% sodium chloride) in 1 : 1 proportion.

The collected blood was centrifuged at 2500 rpm for 10 min to separate the plasma and washed thrice in pyrogen-free normal saline (0.9% w/v) with subsequent centrifugation. The SRBCs were suspended in normal saline, and their count was adjusted to a concentration of 1 × 10^8^ cells in 1 mL of sheep blood which was used as T-lymphocyte-dependent antigens for immunization and challenge of the mice [10, 11].

#### 2.2.4. Acute Oral Toxicity Testing

Female nulliparous and nonpregnant Swiss albino mice were used for the study for each extract and solvent fraction. The test was performed following the Organization for Economic Cooperation and Development (OECD) guideline 425 using limit test method [12].

#### 2.2.5. Animal Grouping and Dosing

SRBC-sensitized and challenged mice were randomly grouped into twelve groups of six mice each that received the respective dose of vehicle/standard drug/extract/solvent fractions as shown in Table 1.

#### 2.2.6. Delayed-Type Hypersensitivity (DTH)

All groups of mice were immunized with 0.1 mL of SRBC (i.p) on day 0 and received an oral dose of respective treatment for 7 days. On the 7th day, one hour after administration of the respective dose, the paw volume of the left leg of each mouse was measured with a digital plethysmometer and challenged with a subcutaneous injection of 0.05 mL of SRBC into the hind footpad of the same leg. The volume of the challenged paw was measured 24 hours after the challenge. The difference in paw volume between the pre- and postchallenge was taken as a measure of DTH response, and the percentage change in paw volume was determined using the following equation [13, 14]:

(1) $$\begin{array}{c} \%\text{ Swlling}=\frac{\left(\text{PV treatment}-\text{PV vehicle}\right)}{\text{PV treatment}}*100, \end{array}$$

PV: paw volume of mice receiving each treatment dose and vehicle.

#### 2.2.7. Effect on Blood Cell Count and Organ Index

At the end of the treatment period of the DTH model, blood was withdrawn from the retro-orbital plexus of all groups of mice to determine blood cells. Following blood withdrawal, the spleen and liver were isolated and weighed immediately to determine their relative index using the following equation [11, 15]:

(2) $$\begin{array}{c} \text{organ index}\left(\text{mg}/\text{g}\right)=\frac{\text{weight of organ}\left(\text{mg}\right)}{\text{weight of mice}\left(\text{g}\right)}. \end{array}$$

### 2.3. Statistical Analysis

The data from each group were analyzed using SPSS version 20 software. Results were presented as mean ± SEM (standard error of mean). The difference of treatment group means from each other and the control mean values was determined by using one-way ANOVA followed by Tukey post hoc multiple comparison test, and *p* values <0.05 were considered significant.

### 2.4. Ethical Consideration

Mice were handled, used, and sacrificed following the procedures approved by the Health Research Ethics Review Committee (HRERC) of Mekelle University, College of Health Sciences, with provided ERC code of 1548/2018.

## 3. Results and Discussion

### 3.1. Acute Oral Toxicity Test

The acute toxicity study showed that no mortality and noticeable behavioral change were observed in all mice that received 2000 mg/kg of the hydroethanolic root extract and solvent fractions (aqueous and *n*-butanol fractions).

### 3.2. Delayed-Type Hypersensitivity Test

In DTH test, groups treated with levamisole, hydroethanolic root extract, and the solvent fraction of *C. adenocaule* showed a dose-dependent increase in DTH response compared to the vehicle (Table 2). This enhancement in DTH response indicated the stimulation of cell-mediated response to SRBC which could be due to the activation of the production of lymphocytes and accessory cell types which play the central role in DTH response. It might also be due to the production of memory T lymphocytes during sensitization with SRBC that are activated to lymphoblasts upon challenging [16]. Hydroethanolic root extract at a dose of 400 mg/kg revealed the highest (0.123 ± 0.010) and statistically significant (*p* < 0.05) DTH response enhancing the paw volume by 47.15% compared to the vehicle. Although the hydroethanolic root extracts at all doses revealed the highest DTH response compared to the solvent fractions of comparable dose, no statically significant difference (*p* > 0.05) was observed. The increased DTH response might be attributable to the presence of tannins, flavonoids, saponins, alkaloids, and carbohydrates [17–19].

### 3.3. Organ Index

The hydroethanolic root extract and solvent fractions of *C. adenocaule* induced an increase in organ index compared to the vehicle (Table 2) which might be attributable to the stimulatory effect of treatment on the growth and development of organs. Hydroethanolic root extract at a dose of 400 mg/kg induced the highest spleen index (5.359 ± 0.174). The increase in organ index might also be due to the stimulatory effect of treatment on proliferation and differentiation of bone marrow stem cells that migrate to and settle within the organ for interaction with the incoming antigen, thereby increasing the mass of the organ [20].

### 3.4. Blood Cell Count

In the present study, Hydroethanolic root extract and solvent fractions of *C. adenocaule* increased the blood cell count compared to the vehicle (Table 3) which might be due to stimulation of the proliferation and differentiation of hematopoietic stem cells (HSCs) in the bone marrow giving rise to functioning blood cells [21]. It might also be due to the reported antioxidant property (IC50 of 38.42 *μ*g/mL) of the study plant that prevents cells from oxidative damage [8]. Hydroethanolic root extract revealed the highest blood cell count compared to solvent fractions of comparable dose which might be accredited to the presence of a larger quantity of antioxidant phytoconstituents at higher concentrations compared to their existence in solvent fractions of comparable dose, thereby enhancing hematopoiesis through synergistic effect [22]. This finding agreed with the finding of Ojogbane and his coworkers who demonstrated the enhancement of WBC count with the administration of aqueous extract of *C. glaucophilla* leaves [23].

## 4. Conclusion

From this study, it can be concluded that hydroethanolic root extract and solvent fractions of *C. adenocaule* had stimulatory properties on the cell-mediated immune response.

## Figures and Tables

**Table 1:** Animal grouping and dosing.

Treatment group dose	Dose
Normal control (vehicle (2% gum acacia))	10 ml/kg
Cyclophosphamide (CP) control	30 mg/kg
Levamisole control	50 mg/kg
Hydroethanolic extract (CEE 100)	100 mg/kg
Hydroethanolic extract (CEE 200)	200 mg/kg
Hydroethanolic extract (CEE 400)	400 mg/kg
Aqueous fraction (AF 100)	100 mg/kg
Aqueous fraction (AF 200)	200 mg/kg
Aqueous fraction (AF 400)	400 mg/kg
*n*-Butanol fraction (BF 100)	100 mg/kg
*n*-Butanol fraction (BF 200)	200 mg/kg
*n*-Butanol fraction (BF 400)	400 mg/kg

**Table 2:** The effect of controls, hydroethanolic root extract, and solvent fractions of *Cyphostemma adenocaule* on delayed-type hypersensitivity response and organ index.

Treatment group	Dose (mg/kg)	DTH (mean ± SEM)	Swelling (%)	Organ index (mg/g)	
				Spleen	Liver
VH	—	0.065 ± 0.013	—	4.594 ± 0.123	44.35 ± 0.862
LEV	50	0.112 ± 0.013^c1^	↑41.96	5.016 ± 0.200^c3^	52.000 ± 1.718^a3c3^
CYP	30	0.055 ± 0.015	↓18.18	3.859 ± 0.094	39.981 ± 0.564
HEE	100	0.091 ± 0.003	↑28.57	4.778 ± 0.107^c3^	48.000 ± 0.807^c3^
	200	0.113 ± 0.011	↑42.48	5.094 ± 0.114^c3^	48.330 ± 1.100^c3^
	400	0.123 ± 0.010^c1^	↑47.15	5.359 ± 0.174^a2c3g1^	52.381 ± 1.720^a3c3^
AF	100	0.081 ± 0.007	↑19.75	4.716 ± 0.128^c3^	47.592 ± 1.122^c3^
	200	0.092 ± 0.005	↑29.35	4.861 ± 0.095^c3^	47.800 ± 1.098^c3^
	400	0.109 ± 0.011^a1c3^	↑40.37	4.967 ± 0.131^c3^	47.912 ± 0.940^c3^
BF	100	0.086 ± 0.013	↑24.42	4.768 ± 099^c3^	47.660 ± 1.202^c3^
	200	0.100 ± 0.007	↑35.00	4.839 ± 0.095^c3^	48.040 ± 1.006^c3^
	400	0.113 ± 0.012^c1^	↑42.48	5.346 ± 0.164^a2c3^	51.815 ± 1.131^a3c3^

Values are expressed as mean ± SEM, *n* = 6. Abbreviations: DTH = delayed-type hypersensitivity, VH = vehicle (2% gum acacia), LEV = levamisole, CYP = cyclophosphamide, HEE = hydroethanolic extract, AF = aqueous fraction, BF = *n*-butanol fraction, ↑ = increase in paw volume, ↓ = decrease in paw volume, a = compared to vehicle, c = compared to cyclophosphamide, g = compared to 100 mg/kg aqueous fraction, 1=*p* < 0.05,  2=*p* < 0.01,  and 3=*p* < 0.001.

**Table 3:** The effect of controls, hydroethanolic root extract, and solvent fractions of *Cyphostemma adenocaule* on blood cell count.

Group	Dose (mg/kg)	Blood cell count					
		WBC (count ×10^3^/mm^3^)				RBC (count ×10^6^/mm^3^)	PLT (count)
		Total	Differentials				
			Lym	Mon	Gra		
VH	—	4.8367 ± 0.5492	3.5400 ± 0.4469	0.7800 ± 0.879	0.5167 ± 0.0792	9.5467 ± 0.3248	427.6667 ± 62.9929
LEV	50	6.0533 ± 0.4933	4.4600 ± 0.4192	1.0800 ± 0.1352	0.5467 ± 0.1551	10.2180 ± 0.2293	482.5000 ± 47.7317
CYP	30	4.0667 ± 0.4248	2.9333 ± 0.2906	0.7000 ± 0.1592	0.4333 ± 0.0422	9.1500 ± 0.2375	371.0000 ± 4.83046
HEE	100	6.8667 ± 1.0544^c1^	4.6000 ± 0.5465	1.4667 ± 0.4295	0.8000 ± 0.1693	10.4870 ± 0.1265^c1^	480.5000 ± 6.5727
	200	7.4333^c2^ ± 0.2963	4.9500 ± 0.2419^c1^	1.6333 ± 0.2044	0.8500 ± 0.0563	10.7750 ± 0.2124^c2^	490.3333 ± 9.5592^c1^
	400	9.0333 ± 0.0211^a3b1c3g1^	6.1667 ± 0.1667^a2c3g1^	1.9167 ± 0.1327^a1c2^	1.100 ± 0.1414^a1b1c2^	10.950 ± 0.1384^a1c3g1^	562.3333 ± .6897^a1c3g1^
AF	100	5.8750 ± 0.8538	3.9500 ± 0.4660	1.2833 ± 0.3049	0.6417 ± 0.1281	9.8500 ± 0.1088	436.8333 ± 14.5863
	200	6.7000 ± 0.2769	4.5167 ± 0.4308	1.4167 ± 0.1108	0.7667 ± 0.1382	9.9550 ± 0.1938	457.6667 ± 3.3928
	400	7.3333 ± 0.4674^c2^	5.1667 ± 0.4609^c1^	1.4833 ± 0.0946	0.6833 ± 0.0477	10.4433 ± 0.2372	465.0000 ± 14.509
BF	100	6.5333 ± 0.7247	4.5167 ± 0.5636	1.3333 ± 0.2765	0.7333 ± 0.1022	10.3520 ± 0.2122	464.5000 ± 14.0327
	200	6.8833 ± 0.3772^c1^	4.6833 ± 0.3772	1.4167 ± 0.1327	0.7833 ± 0.0910	10.4163 ± 0.2118	473.1667 ± 6.8577
	400	7.9833 ± 1.9104^a2c3^	5.2833 ± 0.3868^c2^	1.7333 ± 0.1256^c1^	0.9667 ± 0.0760^c1^	10.7817 ± 0. 2318^c2^	498.1667 ± 4.4603^c1^

VH = vehicle, LEV = levamisole CYP = cyclophosphamide, HEE = hydroethanolic root extract, AF = aqueous fraction, BF = *n*-butanol fraction, WBC = white blood cell, Lym = lymphocyte, Mon = monocyte, Gra = granulocyte, RBC = red blood cell, PLT = platelet, a = compared to gum acacia, b = compared to levamisole, c = compared to cyclophosphamide, g = compared to 100 mg/kg aqueous fraction, 1=*p* < 0.05,  2=*p* < 0.01,  and 3=*p* < 0.001.

## Data Availability

The data generated and/analyzed during the study will be available from the corresponding author upon reasonable request.
